# Severe Chest Pain in a Young Patient with Behçet's Disease: A Rare Manifestation

**DOI:** 10.1155/2022/6032423

**Published:** 2022-02-25

**Authors:** Steen Reed, Michael McGee, Shanathan Sritharan

**Affiliations:** ^1^Department of Medicine, John Hunter Hospital, Australia; ^2^Hunter Medical Research Institute, Australia; ^3^The University of Newcastle, Australia; ^4^The University of New England, Australia; ^5^Department of Medicine, Tamworth Hospital, Australia; ^6^Department of Cardiology, John Hunter Hospital, Australia

## Abstract

A 32-year-old man with a background of Behçet's disease developed severe chest pain. The onset coincided with an episode of sacroiliitis. The patient was diagnosed with pericarditis and was successfully treated with a combination of anti-inflammatory agents. Pericarditis is a rare manifestation of Behçet's disease.

## 1. Case Presentation

### 1.1. History of Presentation

A 32-year-old man with no prior history of cardiovascular disease presented to the emergency department with a 24 hour history of retrosternal chest pain. The pain was described as sharp, pleuritic with some radiation to the left shoulder. The onset of pain was gradual and occurred after an episode of exercise. Serial troponins, electrocardiogram, and chest X-ray were performed and deemed normal. He was commenced on simple analgesia and discharged following a diagnosis of atypical chest pain with a recommendation of outpatient cardiology review.

**Figure 1:**
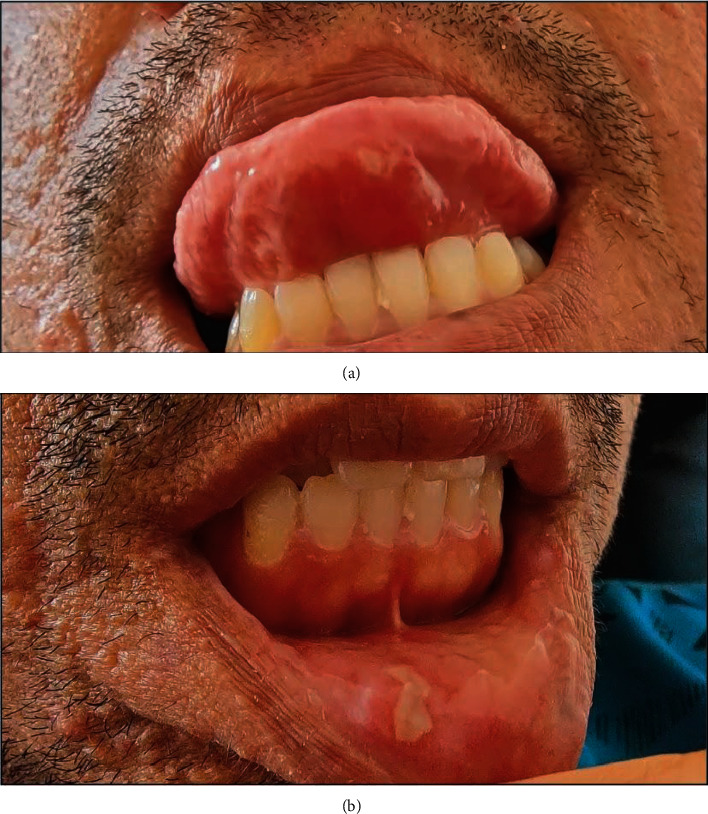
Photographs of the patient. Photographs of the patient's oral mucosa demonstrating aphthous ulcers of the tongue (a) and the lower lip (b).

The patient represented the following day with increasing chest pain despite analgesia. His temperature was 37.9°C, the heart rate 110 bpm, the blood pressure 109/78 mmHg, respiratory rate 16 breaths/min, and oxygen saturation 98% on air. On examination, there were multiple oral aphthous ulcers (Figure 1). There was reproducible tenderness bilaterally on the sacroiliac distraction test. The jugular venous pulse was not elevated. The pulse was regular. Heart sounds were dual with no murmurs and no pericardial rub on auscultation. The lung fields were clear on auscultation. The chest wall was not tender to palpation. There was no peripheral pitting oedema, and there were no clinical signs of deep vein thrombosis.

**Figure 2:**
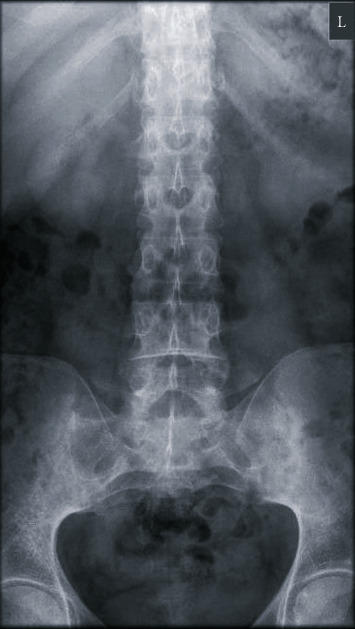
Plain film radiograph of the patient. Anteroposterior plain-film radiograph of the patient performed prior to his initial presentation. Significant sacroiliitis is seen with sclerosis and partial ankylosis of the sacroiliac joints demonstrated bilaterally.

The patient had never experienced chest pain of this nature before. There was no recent history of either respiratory or gastrointestinal viral symptoms. Two days prior to the onset of pain, he was commenced on colchicine 500 *μ*g twice daily for lumbosacral pain attributed to sacroiliitis (Figure 2).

### 1.2. Past Medical History

The patient was diagnosed with Behçet's disease (BD) a year prior to presentation. This was following a history of recurrent oral and scrotal ulcers, associated with self-limiting episodes of colitis. More recently, the patient had been suffering from episodes of large joint polyarthritis and sacroiliitis. Aside from the recent prescription of colchicine for sacroiliitis, the patient's only other medication was paracetamol. The patient had received short courses of oral prednisone to manage symptoms of oral ulcers and arthralgias associated with BD in the recent past. Other past medical history included alpha-thalassemia trait. The patient was of Indian descent. The patient had no personal or family history of tuberculosis and had completed full health screening on immigration to Australia. His family history was significant for cardiovascular disease with his father dying from myocardial infarction at the age of 50.

### 1.3. Investigations

**Figure 3:**
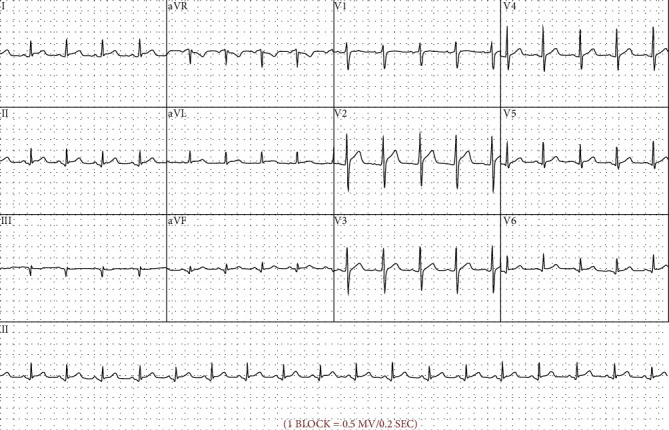
Electrocardiogram of the patient. Electrocardiograph (ECG) recorded during the patient's initial presentation. PR segment depression and ST segment elevation in leads I, II, aVF, aVL, and V2-V6 and demonstrated.

Full blood count revealed white blood cell count 16.7 10^9^/L (range 4.0-11.0 10^9^/L), neutrophil count 12.9 10^9^/L (range 2.0-8.0 10^9^/L), haemoglobin 129 g/L (range 130-180 g/L), C-reactive protein (CRP) 179 mg/L (range < 5 mg/L), and erythrocyte sedimentation rate 71 mm/hr (range 0-15 mm/hr) (Table 1). D-dimer was 0.76 mg/L (range < 50 mg/L). 3 sets of blood cultures were taken and were negative for growth. On the first presentation to the emergency department, serial troponin I was measured at 8 ng/L (normal range < 26 ng/L) and 15 ng/L, respectively. On the subsequent presentation, troponin I was measured at 64 ng/L, then 63 ng/L on 2 hour repeat. Electrocardiography (ECG) revealed ST-segment elevations and associated PR-segment depression in leads I, II, aVF, aVL, and V2-V6 (Figure 3). Transthoracic echocardiogram showed normal ventricular size and systolic function. The left ventricular ejection fraction was 56%. There was no pericardial effusion or restrictive physiology. Computed tomography pulmonary angiogram (CTPA) showed no evidence of pulmonary embolism and no significant airspace consolidation. Computed tomography coronary angiogram (CTCA) and cardiac magnetic resonance imaging (cMRI) were performed a month following discharge from hospital. CTCA revealed plaque within the mid left anterior descending artery involving the origin of the first diagonal branch, resulting in mild stenosis. The pericardium was noted to be unremarkable with normal contour and no effusion. On cMRI, the pericardium was normal in thickness, and no effusion was seen. No abnormality was detected in the pericardial space. There was no increased myocardial signal on T2 imaging and no postgadolinium contrast enhancement on T1 imaging to suggest myocardial involvement.

### 1.4. Differential Diagnosis

The differential diagnosis for this patient included acute coronary syndrome, pulmonary embolism, atypical infection, and pericarditis.

Given the features of low-grade fever, raised inflammatory markers, and widespread ST-segment changes on ECG presenting in a patient with a background of autoimmune disease, a working diagnosis of pericarditis with possible myocardial involvement was made. Important alternative diagnoses were ruled out following CTCA and CTPA. Whilst the echocardiogram showed no evidence of pericardial effusion, this does not rule out a diagnosis of pericarditis. Given that effusion reflects the degree of inflammation in the pericardial space, it is possible that the development of an effusion may have been suppressed by the commencement of colchicine 2 day prior to onset of symptoms.

### 1.5. Management

On admission, the patient was commenced on aspirin 900 mg three times daily; colchicine was continued at 500ug twice daily. The patient was admitted to the coronary care unit and placed on cardiac monitoring. Despite this, the patient's pain remained severe; 50 mg oral prednisone once daily was therefore commenced in addition to the above regimen. The patient was commenced on bisoprolol 2.5 mg once daily and candesartan 4 mg once daily due to the initial suspicion of myocardial involvement. After 3 days of anti-inflammatory treatment, his chest pain subsided. He was discharged home on colchicine and prednisone. The prednisone was weaned slowly under supervision of the patient's rheumatologist. Thiopurine methyltransferase genotyping, hepatitis serology, and Quantiferon Gold assay were performed in case the patient's treatment needed to be escalated to either azathioprine or biological therapy.

## 2. Discussion

BD is a multisystem inflammatory vasculitis of unclear aetiology. Disease is strongly associated with the presence of the HLA-B51 allele. Whilst BD has a global distribution, cases are seen most frequently in populations around the ancient silk road (Turkey has the highest prevalence) and is rare amongst western populations [1].

The most frequent manifestations of BD are oral and genital aphthous ulcers, ocular lesions, and skin lesions. Others include gastrointestinal inflammation, central nervous system lesions, and arthritis. Whilst not fully understood, the underlying pathophysiology of BD is thought to relate to vasculitis, which can affect both large and small vessels [1].

Cardiac manifestations are rare but important to recognise amongst BD sufferers, as mortality is increased in these patients [2, 3]. In one series, cardiac manifestations were seen in 6% of patients (pericarditis accounting for 38.5% of these). Death was observed in 15.4% of these patients, compared with 5.4% of those without cardiac involvement [3]. In the same series, cardiac manifestations occurred more frequently in male patients and less frequently in patients of European descent [3]. Other cardiac manifestations include valvular insufficiency, intracardiac thrombosis, and myocardial infarction [4].

Treatment is empirical and aimed at reducing inflammation. Suitable agents include nonsteroidal anti-inflammatory drugs, colchicine, and corticosteroids [2, 5]. In this case, the patient received a combination of aspirin, colchicine, and prednisone, which led to resolution of symptoms. In severe or resistant disease, immunosuppressant agents such as cyclophosphamide and azathioprine may be considered [6]. Antitumor necrosis factor alpha (TNFa) agents are emerging as a therapeutic option in severe and life-threatening BD. High-quality randomised controlled trials are sparse, and data generally relates to noncardiac manifestations of BD; however, initial data is promising, and this may represent an important treatment option in the event of life-threatening disease resistant to conventional therapies [7, 8].

## 3. Follow-Up

**Figure 4:**
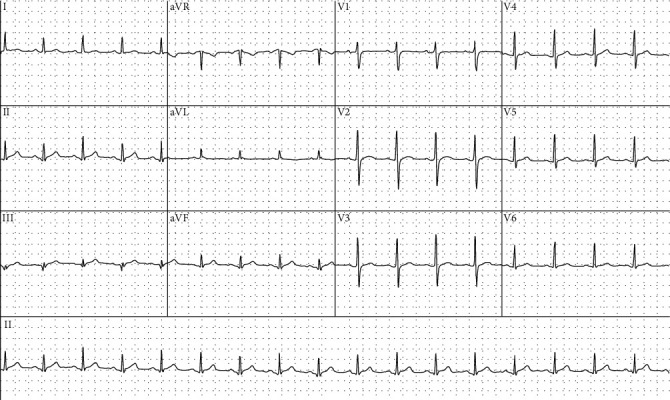
Electrocardiogram of the patient. Electrocardiogram (ECG) recorded during outpatient follow-up in cardiology clinic. This ECG shows resolution of widespread ST-segment elevation following the initial admission with pericarditis.

The patient was followed up by both rheumatology and cardiology in the outpatient setting. The previously noted ECG changes resolved on repeat testing (Figure 4). The patient was continued on regular colchicine, whilst the prednisone was weaned under the supervision of his rheumatologist. The dose of prednisone was weaned to 7.5 mg daily over a period of 8 weeks. Whilst there was no recurrence of symptoms of pericarditis during this time, the patient was suffering from recurrent oral ulcers. Azathioprine was therefore commenced as a steroid sparing therapy.

After cMRI revealed no evidence of myocardial involvement or reduced left ventricular ejection fraction, the candesartan and bisoprolol were ceased. He was commenced on statin therapy due to his family history of ischaemic heart disease and coronary artery atherosclerosis evident on CTCA.

9 weeks after discharge, the patient represented to the hospital with pleuritic chest pain. This occurred 15 days following his second dose of Comirnaty (Pfizer) COVID-19 vaccine. Investigations revealed a CRP of 128 mg/L, normal serial troponin I, and mild ST elevation in inferior and lateral leads on ECG (Table 1). The patient was treated for a recurrence of pericarditis. Prednisone was increased to 25 mg daily with resolution of symptoms. The CRP normalised on repeat testing 2 weeks following this, and the prednisone dose was weaned once more.

## 4. Conclusion

Pericarditis is a rare manifestation of BD. It is important to recognise cardiac manifestations in BD as increased mortality is seen amongst these patients. Pericarditis can be successfully treated with anti-inflammatory agents though immunosuppressants can also be considered. Biological therapy, such as anti-TNFa agents, is a possible treatment option in severe and refractory cases.

## Figures and Tables

**Table 1:** Table summarising the key investigations of the patient.

Investigation	Reference range	1^st^ episode of pericarditis	1 month of anti-inflammatory treatment	2^nd^ episode of pericarditis
CRP	<5 mg/L	179	5	128
ESR	0-15 mm/hr	71	13	51
Troponin I peak	<26 mg/L	64	11	16
WCC	4.0-11.0 10^9^/L	16.7	10.5	13.3
Neutrophil	2.0-8.0 10^9^/L	12.9	6.0	11.2

CRP: C-reactive protein; ESR: erythrocyte sedimentation rate; WCC: white blood cell count.

## Data Availability

The data used to support the findings of this study are available from the corresponding author upon request.
